# Fractional Flow Reserve Derived from Coronary Computed Tomography Angiography Safely Defers Invasive Coronary Angiography in Patients with Stable Coronary Artery Disease

**DOI:** 10.3390/jcm9020604

**Published:** 2020-02-24

**Authors:** Mark Rabbat, Jonathon Leipsic, Jeroen Bax, Brian Kauh, Rina Verma, Demetrios Doukas, Sorcha Allen, Gianluca Pontone, David Wilber, Verghese Mathew, Campbell Rogers, John Lopez

**Affiliations:** 1 Division of Cardiology, Loyola University Medical Center, Maywood, IL 60153, USA; rinaverma1108@gmail.com (R.V.); demetriosdoukas@gmail.com (D.D.); sorchamaryallen@gmail.com (S.A.); dwilber@lumc.edu (D.W.); verghese.mathew@lumc.edu (V.M.); JLOPEZ7@lumc.edu (J.L.); 2 Department of Radiology, St. Paul’s Hospital and the University of British Columbia, Vancouver, BC V6T 1Z4, Canada; JLeipsic@providencehealth.bc.ca; 3 Department of Cardiology, Leiden University Medical Center, Albinusdreef 2, 2333 ZA Leiden, The Netherlands; j.j.bax@lumc.nl; 4 Division of Cardiology, University of Kentucky, Lexington, KY 40536, USA; brian.kauh@gmail.com; 5 Department of Cardiovascular Imaging, Cardiologico Monzino, Via Carlo Parea, 4, 20138 Milan MI, Italy; gianluca.pontone@ccfm.it; 6 HeartFlow, Inc., Redwood City, CA 94063, USA; crogers@heartflow.com

**Keywords:** fractional flow reserve, coronary computed tomography angiography, computational fluid dynamics, stable coronary artery disease

## Abstract

Objectives: In the United States, the real-world feasibility and outcome of using fractional flow reserve from coronary computed tomography angiography (FFR_CT_) is unknown. We sought to determine whether a strategy that combined coronary computed tomography angiography (CTA) and FFR_CT_ could safely reduce the need for invasive coronary angiography (ICA), as compared to coronary CTA alone. Methods: The study included 387 consecutive patients with suspected CAD referred for coronary CTA with selective FFR_CT_ and 44 control patients who underwent CTA alone. Lesions with 30–90% diameter stenoses were considered of indeterminate hemodynamic significance and underwent FFR_CT_. Nadir FFR_CT_ ≤ 0.80 was positive. The rate of patients having ICA, revascularization and major adverse cardiac events were recorded. Results: Using coronary CTA and selective FFR_CT_, 121 patients (32%) had at least one vessel with ≥50% diameter stenosis; 67/121 (55%) patients had at least one vessel with FFR_CT_ ≤ 0.80; 55/121 (45%) underwent ICA; and 34 were revascularized. The proportion of ICA patients undergoing revascularization was 62% (34 of 55). The number of patients with vessels with 30–50% diameter of stenosis was 90 (23%); 28/90 (31%) patients had at least one vessel with FFR_CT_ ≤ 0.80; 8/90 (9%) underwent ICA; and five were revascularized. In our institutional practice, compared to coronary CTA alone, coronary CTA with selective FFR_CT_ reduced the rates of ICA (45% vs. 80%) for those with obstructive CAD. Using coronary CTA with selective FFR_CT_, no major adverse cardiac events occurred over a mean follow-up of 440 days. Conclusion: FFR_CT_ safely deferred ICA in patients with CAD of indeterminate hemodynamic significance. A high proportion of those who underwent ICA were revascularized.

## 1. Introduction

Accurately identifying coronary artery disease (CAD) in patients with symptoms of chest pain is critical in clinical medicine. For nearly four decades, functional-stress testing has served as the standard cardiovascular diagnostic practice for those with stable symptoms suspected to represent CAD, although it has been reported to have low diagnostic yield at the time of invasive coronary angiography (ICA) [1]. A contemporary analysis from the National Cardiovascular Data Registry (NCDR) of more than 385,000 patients from >1100 United States hospitals noted that less than half of patients undergoing exercise-treadmill testing, stress echocardiography, single-photon emission computed tomography (SPECT) imaging, and stress-cardiac magnetic resonance imaging prior to their ICA were found to have obstructive CAD [2,3]. Noninvasive testing made a similar prediction of obstructive CAD compared to clinical factors [2]. In addition, a recent study of over 15,000 patients found that among patients referred for ICA, those with a positive stress test were less likely to have obstructive CAD and receive revascularization compared to those with either a negative stress test or no testing at all [4].

The ideal noninvasive test would identify patients with CAD and lesion-specific ischemia and strengthen the correlation between symptoms and anatomic findings. Coronary computed tomographic angiography (CTA) has emerged as the gold standard noninvasive test for detecting CAD [5,6,7,8]. However, the identification of CAD alone is insufficient, as the relationship between coronary stenosis and ischemia is complex and frequently discordant [9,10,11,12]. In a study of over 1300 coronary artery lesions, 65% of all stenoses with 50–70% diameter reduction and 20% of all stenoses with 71–90% diameter reduction were not hemodynamically significant (FFR ≤ 0.80) [11]. Furthermore, 33% of lesions graded between 31–50% had fractional flow reserve (FFR) values ≤0.80 [12]. FFR is commonly employed to adjudicate lesion-specific ischemia in indeterminate angiographic lesions and to guide revascularization, with its use supported by the guidelines of the European Society of Cardiology and the American Heart/American College of Cardiology [13,14]. Over the past few years, there has been strong interest in computing FFR noninvasively using coronary CTA. The application of computational fluid dynamics (CFD) to resting coronary CTA datasets allows FFR to be calculated noninvasively (FFR_CT_) [15]. The emergence of FFR_CT_ provides a noninvasive test that yields both anatomic and functional data. FFR_CT_ has been validated through a number of accuracy studies and a large clinical utility trial [16,17,18,19,20,21].

There is a paucity of data on the real-world feasibility and outcome of a diagnostic strategy using FFR_CT_ in patients suspected of CAD in the United States. Thus, we sought to determine whether the use of a coronary CTA plus FFR_CT_ guided strategy, as compared to coronary CTA alone, reduces rates of ICA without associated major adverse cardiac events (MACE).

## 2. Methods

Consecutive patients with suspected CAD referred for coronary CTA and FFR_CT_ between May 2015 and June 2017 without known CAD at Loyola University, Chicago (Chicago, IL USA) were included in the analysis. Forty-four patients who underwent CTA alone prior to our institutional implementation of FFR_CT_ were included as controls. Patient demographics and clinical data were collected from the electronic medical records of all patients. The decision to proceed with ICA was at the discretion of the care providers, using information from both the coronary CTA and FFR_CT_ when available. Ineligible patients were defined as those with prior coronary artery bypass graft surgery (CABG), prior percutaneous coronary intervention (PCI), active arrhythmias or acute kidney injury. The study was approved by the Institutional Review Board.

### 2.1. Coronary Computed Tomography Angiography Acquisition and Analysis

Coronary CTA was performed with electrocardiographic-gated prospective or retrospective gating on ≥64 detector row scanners (Siemens Sensation Cardiac 64, Siemens Medical Solutions, Malvern, Pennsylvania, PA, USA; Discovery HD 750, GE Healthcare, Milwaukee, Wisconsin, WI, USA; Revolution CT 256-row, GE Healthcare, Milwaukee, Wisconsin, WI, USA) in accordance with the Society of Cardiovascular Computed Tomography (SCCT) guidelines [22]. Oral, and, when needed, intravenous, beta-blocker was administered to achieve a target heart rate (HR) of 60 bpm. Sublingual nitroglycerin 0.4–0.8 mg was given approximately 5 min prior to contrast administration. CTA datasets were interpreted using a commercially available dedicated workstation (Aquarius 3D Workstation, TeraRecon, San Mateo, CA, USA). Lesions with 30–90% diameter of stenosis were considered of indeterminate hemodynamic significance. Subtotal and total occlusions were classified as ≥90% and 100%, respectively. A coronary lesion with ≥50% diameter of stenosis was considered obstructive on coronary CTA alone. Coronary vessel branches for the left anterior descending, left circumflex, and right coronary arteries were categorized according to the SCCT guidelines [23].

### 2.2. Computation of Fractional Flow Reserve from Coronary Computed Tomography Angiography

FFR_CT_ analysis was performed by HeartFlow Inc. (Redwood City, California, CA, USA) as previously described [15]. After semi-automated segmentation of the epicardial coronary arteries and determination of left ventricular mass, calculations of FFR_CT_ were performed by CFD modeling [15]. Three-dimensional (3D) blood-flow modeling of the coronary arteries was performed, with blood modeled as a Newtonian fluid using incompressible Navier–Stokes equations, and solved subject to appropriate initial and boundary conditions using a finite element method on a parallel supercomputer. As coronary blood flow and pressure were unknown a priori, a technique to couple lumped parameter models of the microcirculation to the outflow boundaries of the 3D model was used [15]. Coronary blood flow was simulated under conditions modeling intravenous adenosine-mediated coronary hyperemia. A positive FFR_CT_ was defined as the nadir value ≤0.80 in a vessel of diameter >1.8 mm. Ongoing clinical studies are evaluating which parameter (nadir value vs. value distal to a lesion) is more appropriate to guide decision-making and yield superior prognostic information [24,25,26,27].

### 2.3. Diagnostic Invasive Coronary Angiography

ICA was performed by board-certified interventional cardiologists following clinical indications and imaging standards set forth by the American College of Cardiology/American Heart Association Task Force on Practice Guidelines and the Society for Cardiac Angiography and Interventions [28]. Decision-making to proceed with ICA was at the discretion of the care providers, using information from both the coronary CTA and FFR_CT_.

### 2.4. Study End Points and Follow-Up

Rates of patients having ICA; revascularization with PCI or CABG; and MACE, defined by cardiovascular death, myocardial infarction or unplanned hospitalizations leading to urgent revascularization, were recorded. Revascularization was considered to be urgent when a patient was admitted to hospital with persistent or increasing symptoms (with or without electrocardiographic changes or elevated biomarker levels) and the revascularization procedure was performed during the same hospitalization.

### 2.5. Statistical Analysis

Baseline characteristics of the selected subjects were calculated and presented as frequencies and percentages for categorical variables and mean ± standard deviation (SD) for continuous variables (Table 1). A comparison of the observed ICA rates to what would be expected based on coronary CTA alone, and the differences in these when FFR_CT_ is available, was performed by analyzing 2 × 2 contingency tables of ICA (Yes/No) and maximum stenosis >/≤ 50% (ICA expected, based on coronary CTA alone) stratified by FFR_CT_ availability/unavailability. Rates within strata were tested using the Fisher’s exact test; differences between FFR_CT_ strata were tested using the Cochran–Mantel–Haenszel χ^2^ test. Also calculated were 95% exact confidence intervals (CI) for ICA rates (Table S1). All analyses were performed using SAS Proprietary software (version 9.2, SAS Institute, Cary, North Carolina, NC, USA).

## 3. Results

A total of 387 stable patients were managed using a coronary CTA/FFR_CT_ diagnostic strategy, whilst 44 underwent coronary CTA alone and served as control patients. The baseline clinical characteristics of patients are shown in Table 1. For those that underwent coronary CTA and selective FFR_CT,_ the mean age was 58.9 ± 13.1 years with a female predominance (51%). Comorbidities included hypertension in 60%, diabetes in 17%, and hyperlipidemia in 64%. Mean BMI was 29.7 kg/m^2^. Approximately half of the patients were referred with atypical chest pain and 149 (39%) patients underwent functional stress testing less than six months prior to CTA acquisition. Using the Diamond–Forrester score, 90.1% of patients were at an intermediate clinic risk.

For those that underwent coronary CTA/FFR_CT,_ 71.2% of patients received metoprolol before the scan, with an average oral dose of 106 ± 57 mg reaching an HR during the scan of 59 ± 7 bpm (Table 2). Sublingual nitroglycerin was administered in all but one patient. The use of beta-blockers and sublingual nitroglycerin was not reported in nine and ten patients, respectively. Mean radiation doses for prospective and retrospective acquisitions were 4.8 mSv and 10.9 mSv, respectively.

Out of the 387 patients, 204 had CAD of indeterminate hemodynamic significance by coronary CTA and were submitted for possible FFR_CT_. FFR_CT_ results were available in 187 of 204 (92%) patients. Coronary CTA image quality was not acceptable for FFR_CT_ analysis in 17 of 204 (8%) patients due to motion artifact, calcification and misregistration.

On coronary CTA, 121 patients (32%) had at least one vessel with ≥50% diameter of stenosis; 67/121 (55%) patients had at least one vessel with nadir FFR_CT_ ≤ 0.80, 55/121 (45%) underwent ICA, and 34 were revascularized (24 PCI, 10 CABG) (Table 3). Of the 21 patients who underwent ICA without receiving revascularization, two were FFR_CT_-negative throughout, seven had distal vessel tip FFR_CT_ values between 0.75–0.80 (gray zone), and four were FFR_CT_-negative 1–2 cm distal to stenoses. The proportion of ICA patients undergoing revascularization was 62% (34 of 55).

On coronary CTA, 90 patients (23%) had vessels with 30–50% diameter of stenosis; 28/90 (31%) patients had at least one vessel with nadir FFR_CT_ ≤ 0.80, 8/90 (9%) underwent ICA, and five were revascularized (4 PCI, 1 CABG). Of the three patients who underwent ICA without receiving revascularization, one was FFR_CT_-negative throughout, one (1%) had distal vessel tip FFR_CT_ values between 0.75–0.80 and two had FFR_CT_-negative 1–2 cm distal to the stenoses.

For those that underwent coronary CTA alone (control patients), ten patients (23%) had at least one vessel with ≥50% diameter of stenosis; 8/10 (80%) underwent ICA, and three were revascularized (3 PCI, 0 CABG). One patient in the control group experienced unstable angina leading to urgent revascularization three years after their CTA. Based upon what would have been expected in our institutional practice, compared to coronary CTA alone, coronary CTA and selective FFR_CT_ reduced the rates of ICA (45% vs. 80%) for those with obstructive CAD. The proportion of ICA patients undergoing revascularization was 38% (3/8).

Only 1% of patients who had stenosis <50% and were FFR_CT_-negative underwent ICA. Three of the 40 patients (8%) who had stenosis ≥50% and were FFR_CT_-negative underwent ICA. Of the patients with stenosis ≥50% with positive nadir FFR_CT_ 61% underwent ICA (Table 3).

**Figure 1:**
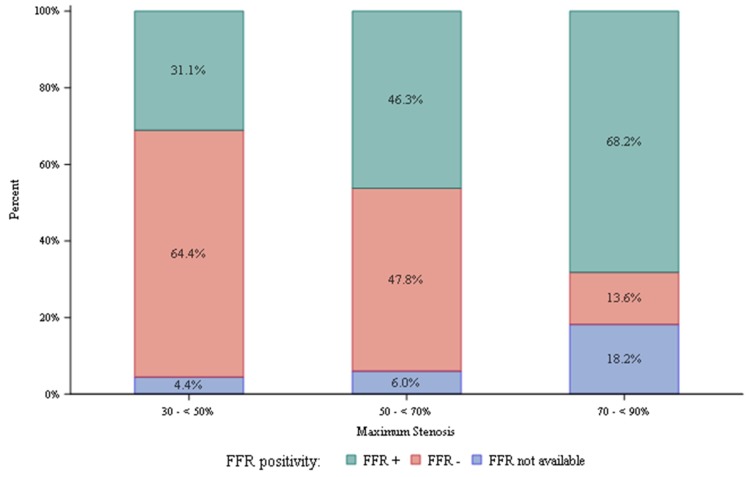
Fractional flow reserve from coronary computed tomography angiography (FFR_CT_) results stratified according to computed tomography angiography stenosis diameter reduction. Nadir FFR_CT_ ≤ 0.80 was positive. Nadir FFR_CT_ > 0.80 was negative.

**Figure 2:**
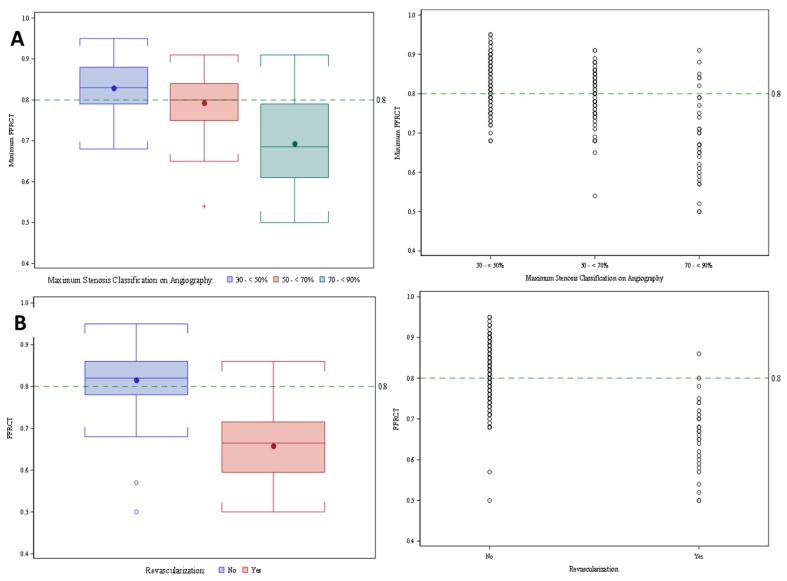
Distribution of diameter stenosis, fractional flow reserve from coronary computed tomography angiography (FFR_CT_) and revascularization. (**A**) Boxplots and scatterplots of FFR_CT_ value by stenosis category. (**B**) Boxplots and scatterplots of FFR_CT_ value by revascularization.

Stratified according to CTA stenosis, 14% of lesions 70–90% diameter of stenosis, 48% of lesions 50–69% diameter of stenosis, and 64% of lesions 30–50% diameter of stenosis were FFR_CT_-negative (Figure 1). ICA and revascularization percentages for those with 70–90%, 50–69%, and 30–49% diameter stenosis were 71, 27, 9 and 52, 12, 6, respectively. Patients who underwent revascularization had significantly lower mean FFR_CT_ values (0.66 vs. 0.83, *p* < 0.0001). Mean FFR_CT_ values for lesions with 30–50%, 50–69% and 70–90% diameter of stenosis were 0.82, 0.79 and 0.69, respectively. Figure 2 represents distributions of diameter stenosis, FFR and revascularization.

**Figure 3:**
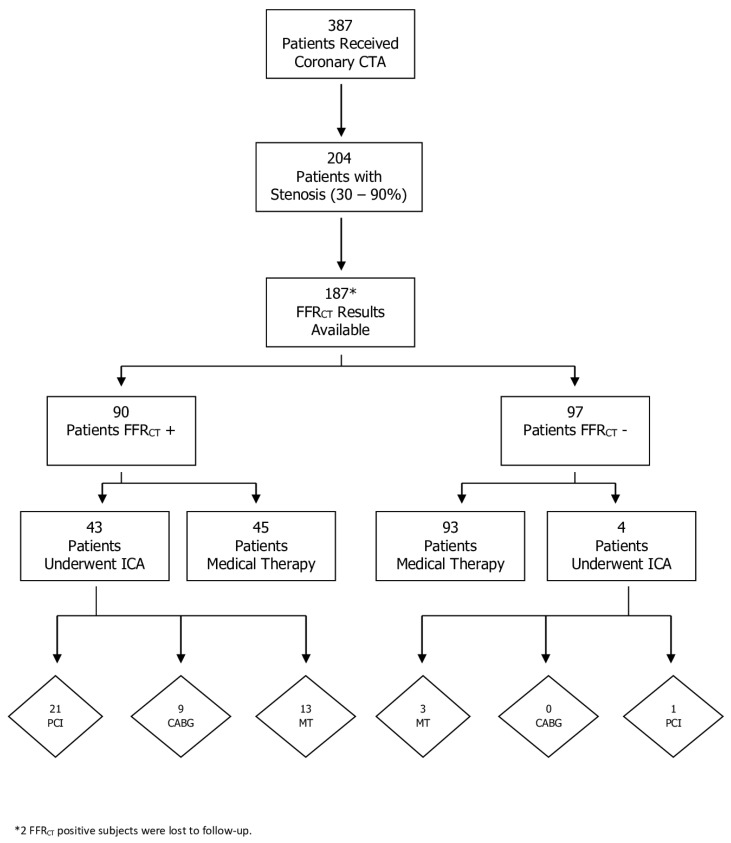
Flowchart for patients with intermediate coronary artery disease and fractional flow reserve from coronary computed tomography angiography (FFR_CT_) availability. CTA, computed tomography angiography datasets; ICA, invasive coronary angiography; PCI, percutaneous coronary intervention; CABG, coronary artery bypass graft surgery; MT, medical therapy.

**Figure 4:**
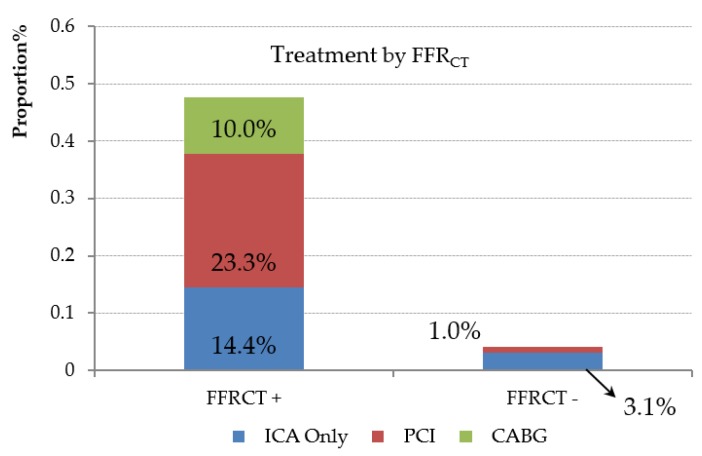
Outcome of invasive coronary angiography (ICA) or revascularization with percutaneous coronary intervention (PCI) or coronary artery bypass graft surgery (CABG) according to fractional flow reserve derived from coronary computed tomographic angiography (FFR_CT_) positivity.

Among those with CAD of indeterminate hemodynamic significance and FFR_CT_ availability, 47 patients underwent ICA and 31 were revascularized (22 PCI, 9 CABG) (Figure 3). Of the 16 patients who underwent ICA without receiving revascularization, three were FFR_CT_-negative. In the remaining 13 patients, six were FFR_CT_-negative 1–2 cm distal to the stenoses and eight of the 13 patients had distal tip FFR_CT_ values between 0.75–0.80. Of patients with a positive nadir FFR_CT_, 50% (45 of 90) did not undergo ICA (Figure 4). The mean distal FFR_CT_ value for all FFR_CT_-positive vessels in these 45 patients was 0.75. One patient with a negative FFR_CT_ underwent revascularization.

**Figure 5:**
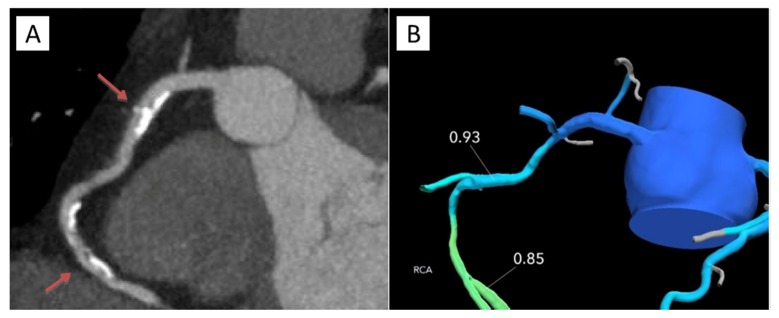
Study patient case. A 48-year-old male with a family history of coronary artery disease, dyspnea on exertion and atypical chest pain underwent coronary CTA. Multiplanar reformat of coronary CTA of the RCA (**A**), and FFR_CT_ (**B**). RCA demonstrated proximal and mid-calcified and non-calcified intermediate (50–70%) stenoses (red arrows) without evidence of lesion-specific ischemia. FFR_CT_ values distal to the proximal and mid RCA stenoses were 0.93 and 0.85, respectively. The patient safely avoided ICA and has been asymptomatic in follow-up on optimal medical therapy. FFR_CT,_ fractional flow reserve derived from coronary computed tomography angiography (CTA) datasets; RCA, right coronary artery; ICA, invasive coronary angiography.

**Figure 6:**
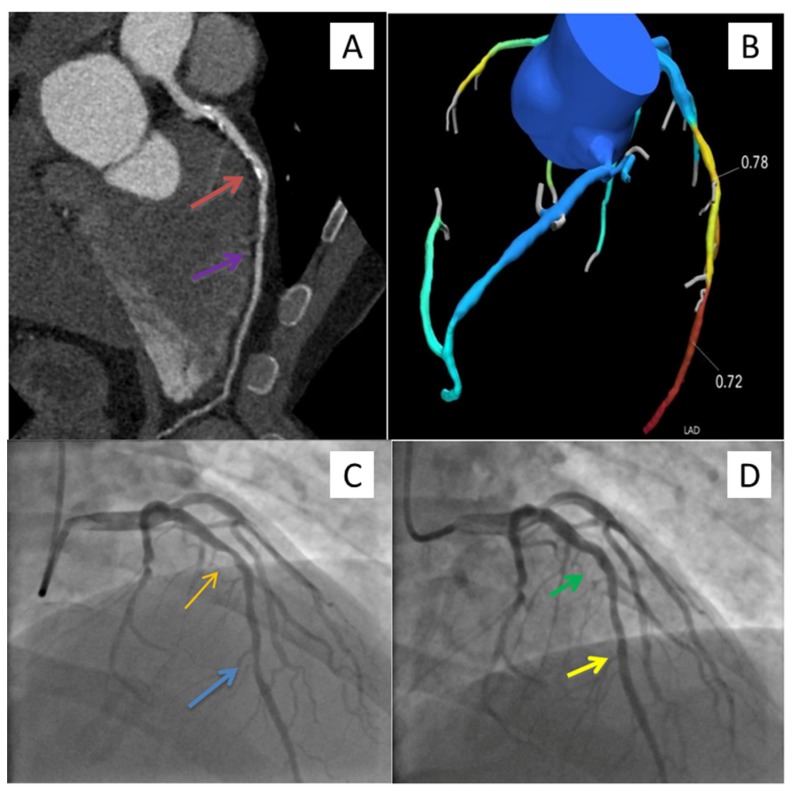
Study patient case. A 48-year-old male with hypertension, diabetes, dyspnea on exertion and atypical chest pain underwent coronary CTA. Multiplanar reformat of coronary CTA of the LAD (**A**), FFR_CT_ (**B**), ICA pre- (**C**) and post- (**D**) PCI. LAD demonstrated a mid-calcified and non-calcified intermediate (50–70%) stenosis and a distal non-calcified intermediate (50–70%) stenosis (red and purple arrows), with evidence of lesion-specific ischemia. FFR_CT_ values distal to the mid and distal LAD stenoses were 0.78 and 0.72, respectively. The patient underwent successful PCI (green and yellow arrows) of the mid and distal LAD stenoses (orange and blue arrows). FFR_CT,_ fractional flow reserve derived from coronary computed tomography angiography (coronary CTA) datasets; ICA, invasive coronary angiogram; LAD, left anterior descending artery; PCI, percutaneous coronary intervention.

For patients who underwent coronary CTA and selective FFR_CT,_ there were no adverse events in a mean follow-up interval of 440 days (range, 135–1277 days). Figure 5 and Figure 6 represent patient cases of non-ischemia and ischemia causing stenoses.

## 4. Discussion

In our evaluation of intermediate clinical follow-up of FFR_CT_ in clinical practice, we identified a number of important findings:

(1) FFR_CT_ was feasible with a conclusive result in >90% of patients;
(2) A diagnostic strategy of coronary CTA plus FFR_CT_ was associated with less ICA in patients with CAD, compared to coronary CTA alone;
(3) Among those who deferred ICA, there was no MACE after more than a one-year follow-up;
(4) A high proportion of those who underwent ICA were revascularized, resulting in higher diagnostic ICA yield and more efficient utilization of catheterization lab resources.

Over the past decade, the field of coronary CTA has seen tremendous progress. Anatomic assessment using coronary CTA is excellent for the detection and exclusion of CAD [5,6,7,8]. Recent studies, such as the SCOT-HEART trial, established that, in patients with suspected angina due to CAD, coronary CTA halved fatal and non-fatal myocardial infarction [29,30]. A contemporary systematic review and meta-analysis of over 20,000 patients determined that, compared with functional-stress testing, coronary CTA was associated with reduced incidence of myocardial infarction, but with an increased incidence of ICA [31]. Coronary CTA alone tends to overestimate the severity of disease, and the relationship between stenosis and ischemia is poor [9,10,11,12]. In the majority of patients with stable ischemic heart disease, a revascularization strategy based only on anatomic evidence of CAD does not appear to confer clinical benefit [32,33,34]. On the other hand, revascularization of functionally significant CAD as assessed by FFR improves clinical outcomes in a highly cost-effective manner [35,36,37,38]. These findings led invasive FFR to become the gold standard test for determining the functional significance of indeterminate lesions and in guiding revascularization, supported by American and European guidelines [13,14]. Recently, there has been great interest in deriving FFR noninvasively, augmenting the anatomic data from coronary CTA with the functional relevance of disease in a lesion-specific manner. FFR_CT_ has been validated in a number of accuracy studies, with the most recent NXT trial reporting per-vessel sensitivities and specificities of 84% and 86%, respectively [16,17,18,20]. Based on the evidence, our diagnostic pathway utilizing coronary CTA and FFR_CT_ when needed is promoted by objective third-party bodies, such as the National Institute for Health and Care Excellence (NICE), whose stable chest pain guidelines recommend coronary CTA in lieu of functional testing as the first-line test for the evaluation of patients with chest pain and FFR_CT,_ as it may avoid the need for ICA [39,40].

FFR_CT_ was feasible with a conclusive result in >90% of patients. This finding is in line with prior studies performed outside the United States [41,42,43]. Clinical interpretation of FFR_CT_ in conjunction with anatomic assessment of CAD by coronary CTA is dependent on appropriate coronary luminal modeling. Inadequate signal or contrast relative to noise and coronary motion or misalignment artifacts may compromise the accuracy of plaque, lumen, CT interpretation and FFR_CT_ analysis. Misalignment artifact has consistently been shown to mostly affect the accuracy of FFR_CT_ [44]. Guideline-directed coronary CTA acquisition methods, including adequate beta-blockade for heart rate control and nitroglycerin, are designed to optimize image quality. Adherence to these methods, in conjunction with feedback on the acceptability of data sets for FFR_CT_ analysis, may improve acceptance rates for FFR_CT_ and coronary CTA image quality, even at experienced centers.

In the invasive arm of the prospective PLATFORM (Prospective LongitudinAl Trial of FFR_CT_: Outcome and Resource Impacts) clinical utility trial, a diagnostic strategy guided by FFR_CT_ resulted in the elimination of 61% of previously planned ICA with no adverse events over a one-year follow-up [20]. In doing so, coronary CTA and FFR_CT_ helped enrich the population undergoing ICA, with an 83% reduction in the incidence of non-obstructive disease noted on ICA. Our findings are in line with PLATFORM, and, in our study, FFR_CT_ reduced the frequency of ICA.

Half (45 of 90) of patients with nadir FFR_CT_ positivity did not proceed with ICA. The mean distal FFR_CT_ value for all FFR_CT_-positive vessels in these 45 patients was 0.75. FFR values between 0.75–0.80 have been described as the “gray zone”, with clinically relevant ischemia ambiguous in this range [45]. Among those with 30–50% diameter of stenosis, a minority of nadir FFR_CT_-positive vessels underwent ICA and revascularization. A recent study found that high-risk plaque, increasing lipid necrotic core and non-calcified plaque burden on coronary CTA predict ischemia in non-obstructive lesions [46]. Thus, for those with 30–50% diameter of stenosis, it may be reasonable to reserve FFR_CT_ for those lesions with adverse plaque characteristics or significant atherosclerotic burden. Further studies are needed to help define the role of FFR_CT_ in stenoses <50%. Additionally, in our practice, the focus when interpreting FFR_CT_ has shifted to FFR_CT_ values that are distal to a treatable focal stenosis. Simply relying on distal-tip values rather than values distal to lesions may not be the most clinically significant [24,25]. Decisions to proceed with ICA should involve additional information, such as anatomy, the location of stenosis, vessel size, suitability for performing revascularization, patient symptoms and clinical judgment.

Norgaard et al. recently demonstrated that deferring ICA in patients with FFR_CT_ > 0.80 had a favorable short-term prognosis (median follow-up period of 12 months) [41]. Importantly, in our study, there were no adverse events in a slightly longer follow-up interval of 15 months. This underscores the favorable clinical outcome in individuals with FFR_CT_ > 0.80 and in select patients with distal-tip FFR_CT_ ≤ 0.80, which may further aid clinicians in their decision-making.

Although diagnostic ICA utilization was reduced using the coronary CTA/FFR_CT_ strategy, among those with obstructive CAD, the proportion of ICA patients who underwent revascularization was 62% (34 of 55). Multiple other studies in various clinical settings, including Emergency Departments, the Veterans Affairs health system, and in various countries, have reported lower diagnostic yield and revascularization rates with a standard of care practice not incorporating FFR_CT_ in the diagnostic pathway [47,48,49,50,51,52]. We observed a higher diagnostic yield of cardiac catheterization through improved patient selection combining anatomic with functional data in one platform using FFR_CT._ This strategy enriched the catheterization-laboratory experience for our patients by sending those individuals to the laboratory who would benefit most from revascularization.

Our study has several limitations. Being an observational study, patients were not randomized and there was inherent subjectivity of patients referred for CTA, FFR_CT_ evaluation, ICA and revascularization. FFR_CT_ was adjudicated positive if the value was below 0.80 anywhere along the length of the vessel. As clinical data were collected from the hospital electronic medical record, there could have been a small number of patients who had follow-up at another health system which were not accounted for. Prior to our institutional implementation of FFR_CT_, we performed very few coronary CTAs. Thus, our control group is small. Finally, the generalizability of the study is limited as the data is from a single center with access to FFR_CT_.

## 5. Conclusions

FFR_CT_ is feasible and has utility within the United States healthcare system. Deferral of ICA based on coronary CTA and FFR_CT_ is safe and improves catheterization-laboratory efficiency.

## Figures and Tables

**Table 1:** Baseline patient characteristics.

Characteristic	Coronary CTA + FFR_CT_ (*n* = 387)	Coronary CTA (*n* = 44)
Age	58.9 (13.1)	59 (10)
BMI (kg/m^2^)	29.7 (6.0)	28.9 (7.6)
Male	190 (49.2)	18 (41.0)
Diabetes Mellitus	63 (16.5)	8 (18.2)
Hyperlipidemia	244 (64.0)	24 (54.5)
Hypertension	229 (60.1)	24 (54.5)
Smoker		
Current	42 (11.1)	8 (18.2)
Ex	128 (33.8)	7 (15)
Never	209 (55.2)	29 (66.8)
Anginal Typicality		
Asymptomatic	52 (13.7)	4 (9)
Atypical	189 (49.7)	32 (72.7)
Non-anginal	102 (26.8)	1 (2.3)
Typical	37 (9.7)	7 (16)
Prior Functional Stress Test	149 (38.5)	29 (65.9)
Diamond Forrester Score		
Low	13 (5.1)	6 (13.6)
Intermediate	228 (90.1)	37 (84.1)
High	12 (4.7)	1 (2.3)
Pre-CTA Aspirin	125 (32.8)	13 (29.5)
Pre-CTA Statin	188 (49.3)	18 (41)
Pre-CTA Beta-blocker	95 (24.9)	13 (29.5)
Pre-CTA Calcium channel blocker	60 (15.8)	8 (18.2)
Pre-CTA ACEi	78 (20.5)	4 (9.1)
Pre-CTA ARB	63 (16.5)	5 (11.4)
Pre-CTA Thiazide	80 (21)	7 (15.9)
Pre-CTA Nitrate	2 (0.5)	1 (2.3)

Data are expressed as mean ± SD or number (%) of patients. CTA, computed tomography angiography; FFR_CT_, fractional flow reserve from coronary computed tomography angiography; BMI, body mass index; ACE-I, angiotensin-converting enzyme inhibitors; ARB, angiotensin receptor blockers.

**Table 2:** Coronary CTA acquisition characteristics.

Acquisition Characteristic	Coronary CTA
Heart Rate, bpm; Mean ± SD (Range)	59 ± 7 (40–80)
Pre-scan administration of nitrates	376 (99.7%)
Pre-scan administration of beta-blockers	269 (71.2%)
Prospective acquisition	42 (10.9%)
Retrospective acquisition	345 (89.1%)
Effective CTA radiation dose, mSv	
Prospective acquisition	4.8 ± 1.8
Retrospective acquisition	10.9 ± 6.0

Values are mean ± standard deviation, range, or *n* (%). CTA, computed tomography angiography.

**Table 3:** Outcome of ICA and revascularization based on CT stenosis and FFR_CT._

FFR_CT_	Stenosis	*n*	ICA (%)	PCI (%)	CABG (%)	Revascularization (%)
Not available	≥50%	14	11 (79)	5 (36)	1 (7)	6 (43)
	<50%	14	3 (21)	1 (7)	1 (7)	2 (14)
≤0.80	≥50%	67	41 (61)	18 (27)	9 (13)	27 (40)
	<50%	59	5 (9)	3 (5)	0 (0)	3 (5)
>0.80	≥50%	40	3 (8)	1 (3)	0 (0)	1 (3)
	<50%	190	2 (1)	0 (0)	(0)	(0)
Total		384	65 (17)	28 (7)	11 (3)	39 (10)

ICA, invasive coronary angiography; PCI, percutaneous coronary intervention; CABG, coronary artery bypass graft surgery; FFR_CT_ indicates fractional flow reserve derived from coronary computed tomography angiography (CTA) datasets. Three patients were lost to follow-up.

## Supplementary Materials

The following are available online at https://www.mdpi.com/2077-0383/9/2/604/s1, Table S1: ICA Rates by Maximum Stenosis Classification on CTA Stratified by Available FFR_CT_.

## Abbreviations

BMI	body mass index
CAD	coronary artery disease
CTA	computed tomography angiography
FFR	fractional flow reserve
FFR_CT_	fractional flow reserve derived from coronary computed tomography angiography datasets
ICA	invasive coronary angiogram
MACE	major adverse cardiac event
SPECT	single photon emission computed tomography

## References

[1] Patel M.R., Peterson E.D., Dai D., Brennan J.M., Redberg R.F., Anderson H.V., Brindis R.G., Douglas P.S. (2010). Low diagnostic yield of elective coronary angiography. N. Engl. J. Med..

[2] Patel M.R., Dai D., Hernandez A.F., Douglas P.S., Messenger J., Garratt K.N., Maddox T.M., Peterson E.D., Roe M.T. (2014). Prevalence and predictors of nonobstructive coronary artery disease identified with coronary angiography in contemporary clinical practice. Am. Heart J..

[3] Cury R.C. (2014). President’s page: Coronary CT angiography as a gatekeeper to the catheterization laboratory. J. Cardiovasc. Comput. Tomogr..

[4] Vavalle J.P., Shen L., Broderick S., Shaw L.K., Douglas P.S. (2016). Effect of the Presence and Type of Angina on Cardiovascular Events in Patients Without Known Coronary Artery Disease Referred for Elective Coronary Angiography. JAMA Cardiol..

[5] Budoff M.J., Dowe D., Jollis J.G., Gitter M., Sutherland J., Halamert E., Scherer M., Bellinger R., Martin A., Benton R. (2008). Diagnostic performance of 64-multidetector row coronary computed tomographic angiography for evaluation of coronary artery stenosis in individuals without known coronary artery disease: Results from the prospective multicenter ACCURACY (Assessment by Coro. J. Am. Coll. Cardiol..

[6] Meijboom W.B., Meijs M.F.L., Schuijf J.D., Cramer M.J., Mollet N.R., van Mieghem C.A.G., Nieman K., van Werkhoven J.M., Pundziute G., Weustink A.C. (2008). Diagnostic accuracy of 64-slice computed tomography coronary angiography: A prospective, multicenter, multivendor study. J. Am. Coll. Cardiol..

[7] Miller J.M., Rochitte C.E., Dewey M., Arbab-Zadeh A., Niinuma H., Gottlieb I., Paul N., Clouse M.E., Shapiro E.P., Hoe J. (2008). Diagnostic performance of coronary angiography by 64-row CT. N. Engl. J. Med..

[8] Neglia D., Rovai D., Caselli C., Pietila M., Teresinska A., Aguadé-Bruix S., Pizzi M.N., Todiere G., Gimelli A., Schroeder S. (2015). Detection of significant coronary artery disease by noninvasive anatomical and functional imaging. Circ. Cardiovasc. Imaging.

[9] Toth G., Hamilos M., Pyxaras S., Mangiacapra F., Nelis O., De Vroey F., Di Serafino L., Muller O., Van Mieghem C., Wyffels E. (2014). Evolving concepts of angiogram: Fractional flow reserve discordances in 4000 coronary stenoses. Eur. Heart J..

[10] Meijboom W.B., Van Mieghem C.A.G., van Pelt N., Weustink A., Pugliese F., Mollet N.R., Boersma E., Regar E., van Geuns T., de Jaegere P. (2008). Comprehensive Assessment of Coronary Artery Stenoses. J. Am. Coll. Cardiol..

[11] Tonino P.A.L., Fearon W.F., De Bruyne B., Oldroyd K.G., Leesar M.A., Ver Lee P.N., Maccarthy P.A., Van’t Veer M., Pijls N.H. (2010). Angiographic versus functional severity of coronary artery stenoses in the FAME study fractional flow reserve versus angiography in multivessel evaluation. J. Am. Coll. Cardiol..

[12] Curzen N., Rana O., Nicholas Z., Golledge P., Zaman A., Oldroyd K., Hanratty C., Banning A., Wheatcroft S., Hobson A. (2014). Does routine pressure wire assessment influence management strategy at coronary angiography for diagnosis of chest pain? The RIPCORD study. Circ. Cardiovasc. Interv..

[13] Windecker S., Kolh P., Alfonso F., Collet J.-P., Cremer J., Falk V., Filippatos G., Hamm C., Head S.J., Juni P. (2014). 2014 ESC/EACTS Guidelines on myocardial revascularization: The Task Force on Myocardial Revascularization of the European Society of Cardiology (ESC) and the European Association for Cardio-Thoracic Surgery (EACTS) developed with the special contribution of the European Association of Percutaneous Cardiovascular Interventions (EAPCI). Eur. Heart J..

[14] Fihn S.D., Gardin J.M., Abrams J., Berra K., Blankenship J.C., Dallas A.P. (2012). 2012 ACCF/AHA/ACP/AATS/PCNA/SCAI/STS Guideline for the diagnosis and management of patients with stable ischemic heart disease: A report of the American College of Cardiology Foundation/American Heart Association Task Force on Practice Guidelines, and the American College of Physicians, American Association for Thoracic Surgery, Preventive Cardiovascular Nurses Association, Society for Cardiovascular Angiography and Interventions, and Society of Thoracic Surgeons. J. Am. Coll. Cardiol..

[15] Taylor C.A., Fonte T.A., Min J.K. (2013). Computational fluid dynamics applied to cardiac computed tomography for noninvasive quantification of fractional flow reserve: Scientific basis. J. Am. Coll. Cardiol..

[16] Koo B.-K., Erglis A., Doh J.-H., Daniels D.V., Jegere S., Kim H.-S., Dunning A., DeFrance T., Lansky A., Leipsic J. (2011). Diagnosis of ischemia-causing coronary stenoses by noninvasive fractional flow reserve computed from coronary computed tomographic angiograms. Results from the prospective multicenter DISCOVER-FLOW (Diagnosis of Ischemia-Causing Stenoses Obtained Via Noni. J. Am. Coll. Cardiol..

[17] Min J.K., Leipsic J., Pencina M.J., Berman D.S., Koo B.-K., van Mieghem C., Erglis A., Lin F.Y., Dunning A.M., Apruzzese P. (2012). Diagnostic accuracy of fractional flow reserve from anatomic CT angiography. JAMA.

[18] Nørgaard B.L., Leipsic J., Gaur S., Seneviratne S., Ko B.S., Ito H., Jensen J.M., Mauri L., De Bruyne B., Bezerra H. (2014). Diagnostic performance of noninvasive fractional flow reserve derived from coronary computed tomography angiography in suspected coronary artery disease: The NXT trial (Analysis of Coronary Blood Flow Using CT Angiography: Next Steps). J. Am. Coll. Cardiol..

[19] Douglas P.S., Pontone G., Hlatky M.A., Patel M.R., Norgaard B.L., Byrne R.A., Curzen N., Purcell I., Gutberlet M., Rioufol G. (2015). Clinical outcomes of fractional flow reserve by computed tomographic angiography-guided diagnostic strategies vs. usual care in patients with suspected coronary artery disease: The prospective longitudinal trial of FFR(CT): Outcome and resource impacts study. Eur. Heart J..

[20] Douglas P.S., De Bruyne B., Pontone G., Patel M.R., Norgaard B.L., Byrne R.A., Curzen N., Purcell I., Gutberlet M., Rioufol G. (2016). 1-Year Outcomes of FFRCT-Guided Care in Patients With Suspected Coronary Disease: The PLATFORM Study. J. Am. Coll. Cardiol..

[21] Hlatky M.A., De Bruyne B., Pontone G., Patel M.R., Norgaard B.L., Byrne R.A., Curzen N., Purcell I., Gutberlet M., Rioufol G. (2015). Quality-of-Life and Economic Outcomes of Assessing Fractional Flow Reserve With Computed Tomography Angiography: PLATFORM. J. Am. Coll. Cardiol..

[22] Abbara S., Blanke P., Maroules C.D., Cheezum M., Choi A.D., Han B.K., Marwan M., Naoum C., Norgaard B.L., Rubinshtein R. (2016). SCCT guidelines for the performance and acquisition of coronary computed tomographic angiography: A report of the society of Cardiovascular Computed Tomography Guidelines Committee: Endorsed by the North American Society for Cardiovascular Imaging (NASCI). J. Cardiovasc. Comput. Tomogr..

[23] Leipsic J., Abbara S., Achenbach S., Cury R., Earls J.P., Mancini G.J., Nieman K., Pontone G., Raff G.L. (2014). SCCT guidelines for the interpretation and reporting of coronary CT angiography: A report of the Society of Cardiovascular Computed Tomography Guidelines Committee. J. Cardiovasc. Comput. Tomogr..

[24] Rabbat M.G., Berman D.S., Kern M., Raff G., Chinnaiyan K., Koweek L., Shaw L.J., Blanke P., Scherer M., Jensen J.M. (2017). Interpreting results of coronary computed tomography angiography-derived fractional flow reserve in clinical practice. J. Cardiovasc. Comput. Tomogr..

[25] Kueh S.H., Mooney J., Ohana M., Kim U., Blanke P., Grover R., Sellers S., Ellis J., Murphy D., Haque C. (2017). Fractional flow reserve derived from coronary computed tomography angiography reclassification rate using value distal to lesion compared to lowest value. J. Cardiovasc. Comput. Tomogr..

[26] Chinnaiyan K.M., Akasaka T., Amano T., Bax J.J., Blanke P., De Bruyne B., Kawasaki T., Leipsic J., Matsuo H., Morino Y. (2017). Rationale, design and goals of the HeartFlow assessing diagnostic value of non-invasive FFR_CT_ in Coronary Care (ADVANCE) registry. J. Cardiovasc. Comput. Tomogr..

[27] Ball C., Pontone G., Rabbat M. (2018). Fractional Flow Reserve Derived from Coronary Computed Tomography Angiography Datasets: The Next Frontier in Noninvasive Assessment of Coronary Artery Disease. Biomed. Res. Int..

[28] Levine G.N., Bates E.R., Blankenship J.C., Bailey S.R., Bittl J.A., Cercek B., Chambers C.E., Ellis S.G., Guyton R.A., Hollenberg S.M. (2012). 2011 ACCF/AHA/SCAI Guideline for Percutaneous Coronary Intervention. A report of the American College of Cardiology Foundation/American Heart Association Task Force on Practice Guidelines and the Society for Cardiovascular Angiography and Interventions. J. Am. Coll. Cardiol..

[29] Williams M.C., Hunter A., Shah A.S.V., Assi V., Lewis S., Smith J., Berry C., Boon N.A., Clark E., Flather M. (2016). Use of Coronary Computed Tomographic Angiography to Guide Management of Patients With Coronary Disease. J. Am. Coll. Cardiol..

[30] Newby D.E., Adamson P.D., Berry C., Boon N.A., Dweck M.R., Flather M., Forbes J., Hunter A., Lewis S., MacLean S. (2018). Coronary CT Angiography and 5-Year Risk of Myocardial Infarction. N. Engl. J. Med..

[31] Foy A.J., Dhruva S.S., Peterson B., Mandrola J.M., Morgan D.J., Redberg R.F. (2017). Coronary Computed Tomography Angiography vs Functional Stress Testing for Patients with Suspected Coronary Artery Disease: A Systematic Review and Meta-analysis. JAMA Intern. Med..

[32] Boden W.E., O’Rourke R.A., Teo K.K., Hartigan P.M., Maron D.J., Kostuk W.J., Knudtson M., Dada M., Casperson P., Harris C.L. (2007). Optimal medical therapy with or without PCI for stable coronary disease. N. Engl. J. Med..

[33] Frye R.L., August P., Brooks M.M., Hardison R.M., Kelsey S.F., MacGregor J.M., Orchard T.J., Chaitman B.R., Genuth S.M., BARI 2D Study Group (2009). A randomized trial of therapies for type 2 diabetes and coronary artery disease. N. Engl. J. Med..

[34] Al-Lamee R., Thompson D., Dehbi H.-M., Sen S., Tang K., Davies J., Keeble T., Mielewczik M., Kaprielian R., Malik I.S. (2018). Percutaneous coronary intervention in stable angina (ORBITA): A double-blind, randomised controlled trial. Lancet.

[35] Zimmermann F.M., Ferrara A., Johnson N.P., van Nunen L.X., Escaned J., Albertsson P., Erbel R., Legrand V., Gwon H., Remkes W. (2015). Deferral vs. performance of percutaneous coronary intervention of functionally non-significant coronary stenosis: 15-year follow-up of the DEFER trial. Eur. Heart J..

[36] Tonino P.A.L., De Bruyne B., Pijls N.H.J., Siebert U., Ikeno F., Veer M.V., Klauss V., Manoharan G., Engstrom T., Oldroyd K.G. (2009). Fractional flow reserve versus angiography for guiding percutaneous coronary intervention. N. Engl. J. Med..

[37] De Bruyne B., Pijls N.H.J., Kalesan B., Barbato E., Tonino P.A.L., Piroth Z., Jagic N., Mobius-Winkler S., Rioufol G., Witt N. (2012). Fractional flow reserve-guided PCI versus medical therapy in stable coronary disease. N. Engl. J. Med..

[38] Fearon W.F., Nishi T., De Bruyne B., Boothroyd D.B., Barbato E., Tonino P., Juni P., Pijls N., Hlatky M. (2017). Clinical Outcomes and Cost-Effectiveness of Fractional Flow Reserve-Guided Percutaneous Coronary Intervention in Patients With Stable Coronary Artery Disease: Three-Year Follow-Up of the FAME 2 Trial (Fractional Flow Reserve Versus Angiography for Multive. Circulation.

[39] Timmis A., Roobottom C.A. (2017). National Institute for Health and Care Excellence updates the stable chest pain guideline with radical changes to the diagnostic paradigm. Heart.

[40] (2017). Coronary Computed Tomography Angiography with Selective Noninvasive Fractional Flow Reserve. https://app.evidencestreet.com.

[41] Nørgaard B.L., Hjort J., Gaur S., Hansson N., Bøtker H.E., Leipsic J., Mathiassen O.N., Grove E.L., Pedersen K., Christiansen E.H. (2017). Clinical Use of Coronary CTA-Derived FFR for Decision-Making in Stable CAD. JACC Cardiovasc. Imaging.

[42] Nørgaard B.L., Gormsen L.C., Bøtker H.E., Partner E., Nielsen L.H., Mathiassen O.N., Grove E.L., Ovrehus K.A., Gaur S., Leipsic J. (2017). Myocardial Perfusion Imaging Versus Computed Tomography Angiography-Derived Fractional Flow Reserve Testing in Stable Patients With Intermediate-Range Coronary Lesions: Influence on Downstream Diagnostic Workflows and Invasive Angiography Findings. J. Am. Heart Assoc..

[43] Jensen J.M., Bøtker H.E., Mathiassen O.N., Grove E.L., Øvrehus K.A., Pedersen K.B., Terkelsen C.J., Christiansen E.H., Maeng M., Leipsic J. (2017). Computed tomography derived fractional flow reserve testing in stable patients with typical angina pectoris: Influence on downstream rate of invasive coronary angiography. Eur. Heart J. Cardiovasc. Imaging.

[44] Leipsic J., Yang T.-H., Thompson A., Koo B.-K., Mancini G.B.J., Taylor C., Budoff M.J., Park H.B., Berman D.S., Min J.K. (2014). CT angiography (CTA) and diagnostic performance of noninvasive fractional flow reserve: Results from the Determination of Fractional Flow Reserve by Anatomic CTA (DeFACTO) study. Am. J. Roentgenol..

[45] Pijls N.H.J., Sels J.-W.E.M. (2012). Functional Measurement of Coronary Stenosis. J. Am. Coll. Cardiol..

[46] Feuchtner G.M., Barbieri F., Langer C., Beyer C., Widmann G., Friedrich G.J., Cartes-Zumelzu F., Plank F. (2019). Non obstructive high-risk plaque but not calcified by coronary CTA, and the G-score predict ischemia. J. Cardiovasc. Comput. Tomogr..

[47] Bradley S.M., Maddox T.M., Stanislawski M.A., O’Donnell C.I., Grunwald G.K., Tsai T.T., Ho P.M., Peterson E.D., Rumsfeld J.S. (2014). Normal coronary rates for elective angiography in the Veterans Affairs Healthcare System: Insights from the VA CART program (veterans affairs clinical assessment reporting and tracking). J. Am. Coll. Cardiol..

[48] Hermann L.K., Newman D.H., Pleasant W.A., Rojanasarntikul D., Lakoff D., Goldberg S.A., Duvall W.L., Henzlova M.J. (2013). Yield of routine provocative cardiac testing among patients in an emergency department-based chest pain unit. JAMA Intern. Med..

[49] Hwang I.-C., Kim Y.-J., Kim K.-H., Shin D.-H., Lee S.-P., Kim H.-K., Sohn D.W. (2014). Diagnostic yield of coronary angiography in patients with acute chest pain: Role of noninvasive test. Am. J. Emerg. Med..

[50] Ko D.T., Tu J.V., Austin P.C., Wijeysundera H.C., Samadashvili Z., Guo H., Cantor W.J., Hannan E.L. (2013). Prevalence and extent of obstructive coronary artery disease among patients undergoing elective coronary catheterization in New York State and Ontario. JAMA.

[51] Roifman I., Wijeysundera H.C., Austin P.C., Rezai M.R., Wright G.A., Tu J.V. (2017). Comparison of Anatomic and Clinical Outcomes in Patients Undergoing Alternative Initial Noninvasive Testing Strategies for the Diagnosis of Stable Coronary Artery Disease. J. Am. Heart Assoc..

[52] Wijeysundera H.C., Qiu F., Bennell M.C., Natarajan M.K., Cantor W.J., Smith S., Kingsbury K.J., Ko D.T. (2014). Impact of system and physician factors on the detection of obstructive coronary disease with diagnostic angiography in stable ischemic heart disease. Circ. Cardiovasc. Qual. Outcomes.

