# Supplementary material for: Fractional Flow Reserve Derived from Coronary Computed Tomography Angiography Safely Defers Invasive Coronary Angiography in Patients with Stable Coronary Artery Disease
Source: J Clin Med. 2020 Feb 24;9(2):604. doi: 10.3390/jcm9020604 (PMC7074264; doi:10.3390/jcm9020604)
Supplement: Supplementary file 1 [file jcm-09-00604-s001.pdf]

Table S1. ICA Rates by Maximum Stenosis Classification on CTA Stratified by Available FFR_{CT}.

	≤50% (*n* = 263)	>50% (*n* = 121)	Total (*n* = 384)
FFR_{CT} is not available: ICA			
Yes	3 (21.4%)	11 (78.6%)	14 (50.0%)
No	11 (78.6%)	3 (21.4%)	14 (50.0%)
Rate (95% CI)	21.43 (4.66, 50.80)	78.57 (49.20, 95.34)	50.00 (30.65, 69.35)
Fisher's Exact Test *p*-value	0.0070		
CMH χ 2 p-value	<.0001		

	≤50% (*n* = 263)	>50% (*n* = 121)	Total (*n* = 384)
FFR_{CT} is available: ICA			
Yes	7 (2.8%)	44 (41.1%)	51 (14.3%)
No	242 (97.2%)	61 (57.0%)	303 (85.1%)
Missing	0	2 (1.9%)	2 (0.6%)
Rate (95% CI)	2.81 (1.14, 5.71)	41.12 (31.70, 51.05)	14.33 (10.86, 18.40)
Fisher's Exact Test *p*-value	<0.0001		

CI = confidence interval; ICA = invasive coronary angiogram. CMH = Cochran–Mantel–Haenszel.
